# Clinical efficacy of subsensory sacral neuromodulation in adults with faecal incontinence: The SUBSoNIC crossover randomised controlled trial and cohort follow‐up study

**DOI:** 10.1111/codi.70308

**Published:** 2025-11-11

**Authors:** Paul F. Vollebregt, Yan Li Goh, Anil Bagul, Claire Chan, Tom Dudding, Paul Furlong, Shaheen Hamdy, Joanne Haviland, Richard Hooper, James Jones, Eleanor McAlees, Christine Norton, P. Ronan O'Connell, Michael Powar, S. Mark Scott, Natasha Stevens, Kerry Tubby, Sian Worthen, Yuk Lam Wong, Charles H. Knowles

**Affiliations:** ^1^ Centre for Neuroscience, Surgery and Trauma, Faculty of Medicine and Dentistry, Blizard Institute Queen Mary University of London London UK; ^2^ Department of Gastroenterology and Hepatology, Amsterdam Gastroenterology Endocrinology Metabolism Research Institute Amsterdam University Medical Centre Amsterdam The Netherlands; ^3^ Sandwell and West Birmingham NHS Trust Birmingham UK; ^4^ University Hospitals Birmingham NHS Foundation Trust Birmingham UK; ^5^ Pragmatic Clinical Trials Unit, Centre for Evaluation and Methods, Faculty of Medicine and Dentistry, Wolfson Institute of Population Health Queen Mary University of London London UK; ^6^ University Hospital Southampton NHS Foundation Trust Southampton UK; ^7^ Aston Institute of Health and Neurodevelopment, College of Health and Life Sciences Aston University Birmingham UK; ^8^ Centre for GI Sciences, Division of Diabetes, Endocrinology and Gastroenterology University of Manchester Manchester UK; ^9^ Health Sciences Centre, School of Medicine University College Dublin Dublin Ireland; ^10^ Florence Nightingale Faculty of Nursing, Midwifery and Palliative Care King's College London London UK; ^11^ Centre for Colorectal Disease St Vincent's University Hospital Dublin Ireland; ^12^ Cambridge University Hospitals Cambridge UK; ^13^ Cleveland Clinic London London UK

**Keywords:** anal incontinence, faecal incontinence, neuromodulation, sacral nerve stimulation

## Abstract

**Aim:**

Sacral neuromodulation (SNM) is considered the first‐line surgical treatment in adults with refractory faecal incontinence (FI). However, its clinical efficacy has not been rigorously tested in a trial setting.

**Method:**

Randomised, multicentre, double‐blind crossover trial (two 16‐week periods) of active stimulation versus sham, and open‐label follow‐up to 58 weeks. Participants: adults aged 18–80 with refractory FI. Interventions: *Active*: subsensory sacral nerve stimulation with an implanted pulse generator; *Sham*: identical implant but turned off. Primary outcome: FI episodes per week (paper bowel diary) during final 4 weeks of crossover periods (allowing 12 weeks' washout). Randomised allocation (1:1) to arm 1 (SNM/sham) or arm 2 (sham/SNM). Blinding: participants, surgeons, assessors; tamper‐proof tape‐masked stimulation settings. Sample size: 80 patients to detect a 30% reduction in episodes. Groups compared using a paired *t*‐test, and treatment effects summarised by mean differences.

**Results:**

Trial delivery was severely affected by COVID‐19. Thirty‐nine patients of 220 screened (arm 1: *N* = 17; arm 2: *N* = 22) were recruited at 10 sites (February 2018–July 2022), of whom only 16 (arm 1: *N* = 9; arm 2: *N* = 7) had complete primary outcome data. Of the 39, 19 completed follow‐up to 58 weeks. SNM conferred a non‐significant reduction in mean FI episodes per week compared to sham (−0.795 [95% CI: −1.5 to 0.0], *p* = 0.06). Improvements were observed in FI symptoms at 58 weeks compared to baseline (FI episodes per week: 3.2 [SD 3.3] vs. 6.2 [SD 5.9]).

**Conclusions:**

The SUBSoNIC trial failed to find conclusive evidence of the experimental efficacy of SNM. Further demonstration of experimental efficacy remains important as SNM is a high‐cost and invasive therapy.

## INTRODUCTION

Faecal incontinence (FI), defined as the recurrent accidental loss of faecal material leading to a social or hygiene problem [1], is a common condition with a negative impact on quality of life [2] and high societal costs [3]. Treatment of FI starts with conservative management including lifestyle adjustments, pharmacological and behavioural therapies (e.g. pelvic floor physiotherapy ± biofeedback) [3]. Surgical intervention may be offered to those patients with refractory symptoms. Traditional surgical procedures aiming to reconstruct or augment the anal sphincter are only suitable for a minority of patients, have variable success rates and risk morbidity [4]. A stoma is often considered a final option.

Sacral neuromodulation (SNM) is a less invasive treatment option based on chronic low‐amplitude stimulation of the mixed sacral spinal nerves using an implanted multipolar electrode lead and pulse generator. SNM is now considered the first‐line surgical treatment option for adults with FI in whom conservative management has failed [3].

Despite having widespread regulatory approval for FI since 1995 in Europe and 2011 in the United States, current evidence for the efficacy of SNM is based mainly on non‐randomised studies. Such studies, which include those considered pivotal for regulatory approval [5, 6], numerous case series [7] and some national registries [8] consistently show that SNM leads to a substantial reduction in symptoms in the majority of adults with FI undergoing implantation.

By contrast, the evidence base from randomised controlled trials is very limited with only a small number of trials performed, with heterogeneous designs and endpoints [7, 9]. Blinded trials incorporating a sham comparator are even fewer but must be considered the gold standard for separating experimental efficacy (arising from the targeted delivery of electrical charge) from well‐established placebo effects for FI trials that may be greater than 50% based on a recent drug trial [10]. Thus, although SNM is well established from the perspective of clinical effectiveness in practice, it can be argued that an expensive and invasive treatment would still benefit from having greater proof of experimental efficacy. This is particularly relevant since the advent of less expensive and invasive forms of neuromodulation [11, 12]. The objective of this randomised controlled trial and cohort follow‐up study was to determine the clinical efficacy of sub‐sensory chronic low‐amplitude SNM in adults with FI in whom conservative treatment has failed.

## METHODS

A detailed description of the study protocol has been published previously [13].

### Trial design

SUBSoNIC was a multicentre, randomised double‐blind crossover trial in which SNM was compared to sham stimulation. The study recruited at nine sites in the United Kingdom and one site in Ireland. After SNM implantation, patients were randomised to two arms: active stimulation [SNM] followed by sham stimulation [SHAM] or SHAM followed by SNM. Both arms had two intervention periods of 16 weeks (T0–T16 and T16–T32). Efficacy outcomes were evaluated during the final 4 weeks of each intervention (T12–T16 and T28–T32), allowing for 12‐week intervention periods before outcome assessment (and therefore adequate washout for patients in the SNM–SHAM arm).

After the crossover phase, patients were followed up for a further 26 weeks. During this open‐label cohort follow‐up phase, stimulation (either sub‐ or supra‐sensory, based on patients' preference) was given as per routine clinical practice. Further outcomes were evaluated at T54–T58 to study the short‐term effectiveness of SNM within the rigour of a clinical trial unit‐monitored prospective study.

### Eligibility criteria

Consecutive patients (18–80 years) with FI were assessed for broad eligibility at the MDT of the participating sites. These patients had already been deemed clinically suitable for SNM based on routine clinical evaluation ratified by pelvic floor multidisciplinary team discussion (as per NHS England guidance) [14]. Inclusion criteria for full study enrolment and randomisation were

1. Fulfilling Rome III and International Consultation on Incontinence criteria of FI (recurrent involuntary loss of faecal material that is a social or hygiene problem and not a consequence of acute diarrhoea) [1, 15];
2. Failure of conservative therapies according to the NICE standard [diet, bowel habit and toilet access addressed; medication (e.g. loperamide), advice on incontinence products, pelvic floor muscle training, biofeedback and transanal irrigation if appropriate] [3];
3. Minimum of eight FI or faecal urgency episodes (including a minimum of four FI episodes) during a 4‐week screening period.
4. Ability to understand written and spoken English.
5. Ability and willingness to give informed consent.

Exclusion criteria were those commonly considered contraindications to the use of SNM [16] and included: known communication between the anal and vaginal tracts, congenital anorectal malformation, rectal surgery (rectopexy/resection) performed <12 months ago (24 months for cancer), full‐thickness rectal prolapse or a high‐grade intussusception (diagnosed with clinical examination ± dynamic imaging as per local policy), chronic inflammatory bowel diseases, chronic constipation with overflow incontinence, structural evacuation disorder based on examination and/or imaging, active perianal sepsis (including pilonidal sinus), defunctioning loop or end stoma in situ, neurological disease (e.g. diabetic neuropathy, multiple sclerosis, Parkinson's disease), current or future need for MR imaging based on clinical history, complete or partial spinal cord injury, bleeding disorders (e.g. haemophiliac, warfarin therapy), pregnancy or intention to become pregnant during the study period and not fit for surgeon‐preferred method of anaesthesia.

### Interventions

#### SNM surgery

Test stimulation (either monopolar temporary wire or quadripolar tined lead based on local practice) took place before randomisation and was not considered a study intervention. Those patients meeting the clinical response during the test phase underwent implantation of the permanent stimulator. A maximum of 14 weeks was allowed between the start of the test phase and implantation of the permanent stimulator. A commercially available medical device (Medtronic Interstim II™) was surgically implanted as per manufacturer's instructions and standardised placement technique [17] to provide chronic low voltage stimulation of the third or fourth sacral nerve root. In brief, fluoroscopic‐aided percutaneous insertion of the lead using a curved stylet and accepting position only when three out of four electrodes provide low voltage (<3 V) contraction of the anal sphincter and pelvic floor ± big toe.

#### Active stimulation (SNM)

For active stimulation (SNM), the clinician used the standard programming settings (frequency: 14 Hz; pulse width: 210 μs). The optimal electrode configuration was determined by increasing the amplitude of stimulation from zero by steps of 0.1 V for each electrode until the sensory threshold was reached. The sensory threshold and location of stimulation were recorded for each electrode. The electrode configuration that achieved sensation closest to the anus/perineum with the lowest amplitude was chosen for the chronic stimulation. Sub‐sensory chronic stimulation was then initiated by reducing the amplitude to a level just below the habituated sensory threshold (for blinding). A re‐programming visit was scheduled 6 weeks after the start of the intervention to assess for suboptimal efficacy or unwanted effects of stimulation. The electrode configuration could be changed if the site of stimulation appeared to be suboptimal.

#### SHAM stimulation (SHAM)

For sham stimulation (SHAM), sensory thresholds were recorded like the description above; however, subsequently the amplitude was then adjusted to 0 V or 0.05 V (the latter required in some participants due to new device handset limitations). As with the active stimulation (SNM) group, a reprogramming visit was scheduled 6 weeks after the start of the intervention. The actions during the reprogramming visit are described in detail in the study protocol [13].

### Outcomes

Baseline data were collected prior to test stimulation. The primary outcome was reduction in FI episodes per week (recorded using a paper bowel diary over a 4‐week period) during active stimulation (SNM) versus sham stimulation (SHAM) during the crossover phase (T12–T16 and T28–T32). Secondary clinical outcomes were recorded at 16, 32 and 58 weeks and included symptoms captured with an e‐event recorder (real‐time recording of episodes of FI, leakage of flatus and urgency without incontinence, see Figure S1) and a panel of summative symptom and impact questionnaires [St Mark's continence score [18], OAB‐Q SF score [19], FI‐QoL score [20], SF‐ICIQ‐B questionnaire [21], EQ‐5D‐5L [22], Likert scale of patient's global impression of treatment success (scale 0–10)], patient perception of active stimulation (SNM) or sham stimulation (SHAM) (blinding success), electrode settings and adverse events.

### Allocation and blinding

Patients were randomised (1:1) to arm 1 (SNM/SHAM) or arm 2 (SHAM/SNM) at the time of implantation of the permanent stimulator using an online randomisation system managed by the Pragmatic Clinical Trials Unit (PCTU) at Queen Mary University of London. Randomisation was stratified by sex and site with block sizes of 4. Members of the research team, statisticians, surgeons who performed the surgical procedure and patients were blinded to the randomisation. Patients were informed of the allocation ratio of 1:1 and that blinding prevented them from knowing in which arm they were participating. Tamper‐proof tape was used to mask stimulation settings on the patient programmer.

### Sample size

The trial was designed to detect a mean 30% reduction in FI episodes between SNM and SHAM, assuming that for an inactive device in a typical patient, the number of FI events in 4 weeks would have an over‐dispersed Poisson distribution with mean 28 and 95% range 7–112, and that this mean varied between individuals with 95% range 14 and 56. This gave a correlation of 0.2 between log(number of events) for the same individual in two different months, and a standard deviation of 0.775 for log(number of events) in each month, consistent with results of trials in similar populations [11]. With 90% power at the 5% significance level with a cross‐over design this required 90 patients (45 per arm), allowing for 10% loss to follow‐up.

### Statistical methods

Baseline characteristics and symptom questionnaires were summarised by sequence (SNM/SHAM vs. SHAM/SNM) using descriptive statistics. Analyses included all patients according to randomised allocation and with available outcome data. There was no imputation of missing data other than for the sensitivity analysis of the primary outcome (described below).

#### Primary outcome

The pre‐specified statistical analysis plan for the primary outcome was to compare SNM and SHAM during the crossover phase using a mixed Poisson regression model for the counts of FI events. However, the Poisson regression models for the count outcomes failed to converge, owing in part to the small patient numbers. Paired *t*‐tests were used instead to compare the counts of FI events between SNM and SHAM, with treatment effects summarised by mean differences in the number of events per week (95% confidence intervals [CI]). For these analyses, only patients with outcome data in both crossover periods could be included. The paired *t*‐test assumes no period effect, as the within‐patient difference in outcomes is calculated regardless of the order of SNM versus SHAM. To assess the effect of incomplete paper diary completion a sensitivity analysis was done by imputing a zero for days when some (but not all) of the count outcomes were left blank.

#### Secondary outcomes

The pre‐specified statistical analysis plan for the secondary outcomes was to use Poisson regression for count outcomes and linear regression for other quantitative outcomes. Unlike the primary outcome, there were no issues with convergence of the regression models for the secondary outcomes (with the exception of e‐recordings for which paired *t*‐tests were adopted instead). Regression analyses included all available data (i.e. not only patients with data in both crossover periods). Outcomes reported on the paper bowel diaries were compared with the e‐recording equivalent to assess agreement using Bland–Altman plots. Data in patients with outcome measurements at baseline and at the end of the study (58 weeks) were summarised using descriptive statistics, with no formal statistical comparison.

Analyses were done using Stata V17.0 (College Station, TX).

## RESULTS

### Participants

**FIGURE 1:**
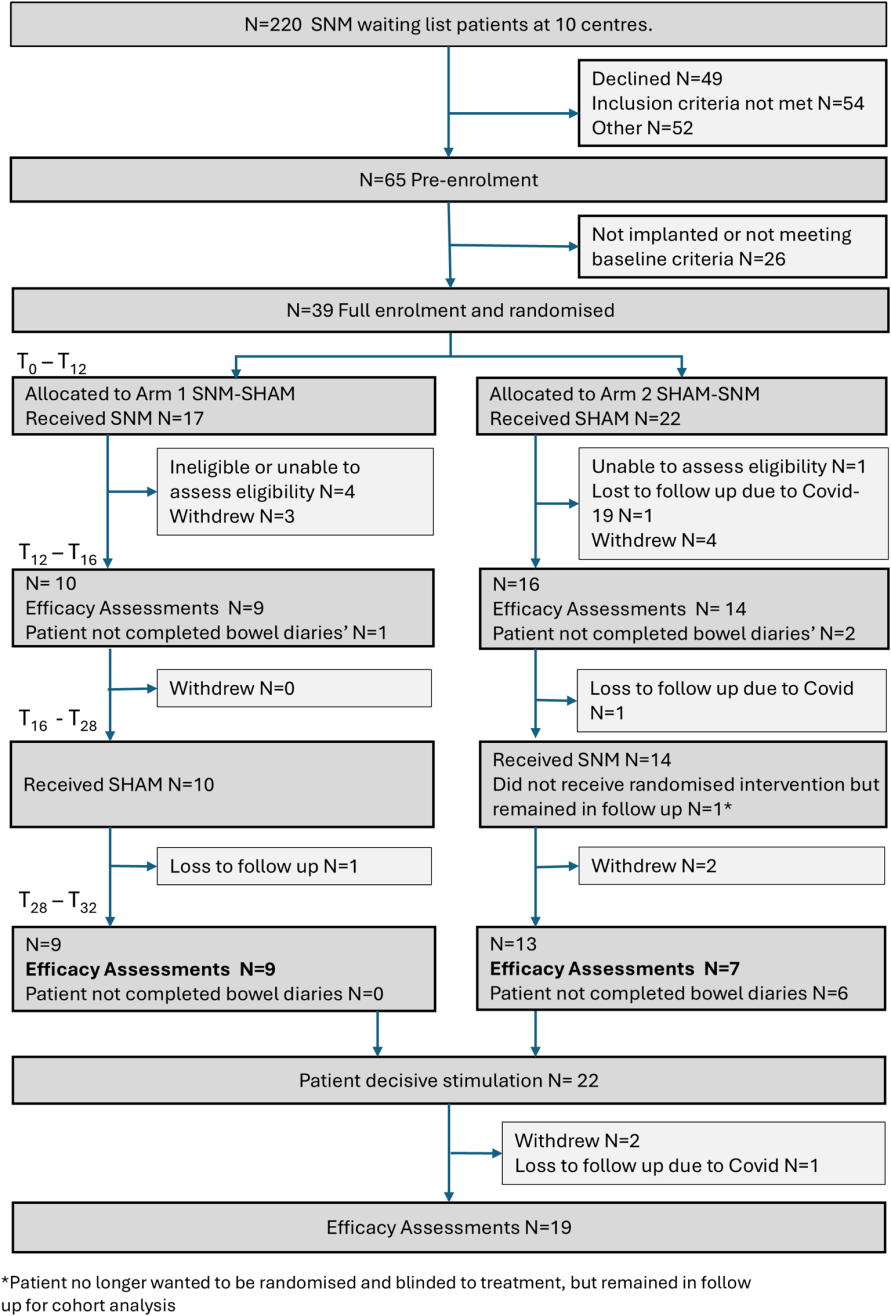
Flow diagram.

A total of 220 patients were screened for eligibility at the 10 participating sites between February 2018 and July 2022 (Figure 1). The trial was terminated early on 24 July 2022 on the advice of the Data Monitoring and Ethics Committee on the basis of futility given the ongoing significant barriers due to COVID‐19, with the main barriers being recruitment, the inability to continue face‐to‐face patient visits, redeployment of research staff to COVID‐19 facing clinical roles and cancelling of SNM surgery due to lack of priority for non‐urgent surgery. Of the 220 patients screened, 155 were ineligible due to study‐specific exclusion criteria or declined study participation (Figure S2). Of the remaining 65 patients pre‐enrolled and consented to the study, 39 patients were randomised (SNM/SHAM: *n* = 17; SHAM/SNM: *n* = 22), of whom 16 completed the primary outcome during both crossover periods (SNM/SHAM: *n* = 9; SHAM/SNM: *n* = 7). Of the other 23 patients, 12 withdrew from the study, 5 were excluded based on eligibility criteria, and 6 did not complete the primary outcome data (these were still included in the cohort follow‐up phase). Specific reasons for withdrawal from the crossover and cohort follow‐up phase are detailed in Table S7. In total, 19 patients completed the cohort follow‐up phase.

### Clinical characteristics

Clinical characteristics at baseline of the 39 patients randomised are detailed in Table 1, with no major differences between the two arms. Mean age was approximately 57 years, with most patients (92.3%) being female. Significant comorbidities and previous surgical procedures were reported in the majority of patients. Gynaecological and obstetric history and clinical examination findings at baseline are reported in Tables S1 and S2. All but one female patient reported obstetric history (median of two vaginal deliveries in both arms).

**TABLE 1:** Key baseline demographic and clinical characteristics.

	Randomised allocation	
	SNM/SHAM *N* = 17^a^	SHAM/SNM *N* = 22^a^
Age (years)	*N* = 17	*N* = 22
Mean (SD)	55.9 (14.1)	58.2 (11.8)
Median (IQR)	58 (44, 66)	63.5 (47, 66)
Sex (%)	*N* = 17	*N* = 22
Male/Female	1 (6)/16 (94)	2 (9)/20 (91)
Ethnicity (%)	*N* = 17	*N* = 22
White	17 (100)	19 (86)
Black	0 (0)	1 (5)
Asian	0 (0)	1 (5)
Mixed	0 (0)	1 (5)
Other	0 (0)	0 (0)
Missing	0	0
BMI (kg/m^2^)	*N* = 17	*N* = 18
Mean (SD)	29.5 (6.84)	28.6 (5.87)
Median (IQR)	28.3 (26.0, 30.2)	27.4 (23.9, 32.8)
Significant medical history (%)	*N* = 17	*N* = 22
No	4 (24)	6 (27)
Yes	13 (76)	16 (73)
Missing	0	0
(If yes) Medical history^b^ (%)	*N* = 13	*N* = 16
Cardiovascular	2/13 (15)	6/16 (38)
Respiratory	2/13 (15)	2/16 (13)
Gastrointestinal	4/13 (31)	7/16 (44)
Metabolic	2/13 (15)	2/15 (13)
Haematological	2/13 (15)	0/15 (0)
Warfarin/Heparin Therapy	0/13 (0)	0/15 (0)
Hepatic	0/13 (0)	0/15 (0)
Renal	0/13 (0)	0/15 (0)
Genitourinary	1/13 (8)	4/15 (27)
Neurological/CNS	1/13 (8)	3/15 (20)
Psychiatric	2/13 (15)	5/15 (33)
Dermatological	1/13 (8)	1/15 (6)
Musculoskeletal	2/13 (15)	6/15 (40)
Any Other	5/13 (39)	1/14 (7)
Significant surgical history (%)	*N* = 17	*N* = 22
No	2 (12)	3 (14)
Yes	15 (94)	19 (86)
Missing	0	0
(If yes) Surgical history^b^ (%)	*N* = 15	*N* = 19
Abdominal	7/15 (47)	8/18 (44)
Urogynaecological	8/15 (53)	13/19 (68)
Proctological and perineal	5/15 (33)	8/19 (42)
Neuromodulation	5/15 (33)	2/18 (11)
Other	5/14 (36)	6/18 (33)
Duration of faecal incontinence symptoms (years)	*N* = 17	*N* = 22
Mean (SD)	9.5 (4.8)	6.5 (4.0)
Median (IQR)	10.0 (6.0, 13.0)	5.5 (4.0, 8.4)
Preceding events (%)	*N* = 17	*N* = 21
No	6 (35)	9 (43)
Yes	11 (65)	12 (57)
Missing	0	1
Faecal incontinence symptoms^b^ (%)		
Urgency	12/13 (92)	15/16 (94)
Passive incontinence	12/17 (71)	19/22 (86)
Urge incontinence	15/17 (88)	21/22 (96)
Flatus incontinence	13/17 (76)	20/22 (91)
Prolapse symptoms^b^ (%)		
Sensation of rectal prolapse	3/17 (18)	3/22 (14)
Sensation of vaginal prolapse (female only)	3/16 (19)	2/20 (10)
Sensation of vaginal bulging (female only)	2/16 (13)	1/20 (5)
Anti‐diarrhoeal medications^b^ (%)		
Loperamide	14/17 (82)	16/22 (73)
Other	4/17 (24)	5/21 (24)
Urinary symptoms history^b^ (%)		
Increased frequency	5/17 (29)	14/21 (67)
Urgency	5/17 (29)	14/22 (64)
Stress incontinence	7/17 (41)	10/22 (45)
Urge incontinence	6/17 (35)	12/22 (55)
Previous faecal incontinence treatments^b^ (%)		
Pelvic floor exercises	16/17 (94)	19/22 (86)
Conservative management	17/17 (100)	22/22 (100)
Biofeedback	9/17 (53)	16/22 (73)
Anal irrigation	4/17 (24)	7/22 (32)
PTNS	8/17 (47)	9/22 (41)
Sphincter repair	3/17 (18)	2/22 (9)

^a^ Percentages calculated excluding missing data.

^b^ Where more than one response is possible, denominators may vary for individual items due to missing data.

### Symptoms at baseline

Mean numbers of FI episodes per week at baseline (data collected prior to test stimulation: staged 68%; PNE 32%) for the 39 randomised patients were concordant with trial design assumptions (SNM/SHAM group: mean 6.6 [SD 6.6]; SHAM/SNM group: mean 7.1 [SD 7.8]; Table 2). Other symptoms were similar between the two arms except for the number of urgency episodes per week (without incontinence) and the number of episodes of flatus leakage which were higher in the SHAM/SNM arm. Baseline e‐event recordings were only available in a small number of patients (14/39). Other symptom and quality of life measures at baseline are detailed in Table S3. Median St Mark's incontinence score was approximately 19 in both arms indicating severe symptoms of FI.

**TABLE 2:** Symptoms (bowel diaries) at baseline and during crossover.

	Baseline^a^		T28–T32 (crossover)^b^			
	SNM/SHAM *N* = 13	SHAM/SNM *N* = 20	SNM *N* = 16	SHAM *N* = 16	Mean difference^c^ (95% CI)	*p* ^d^
Primary outcome						
Number of FI episodes per week (urge + passive)						
Mean (SD)	6.6 (6.6)	7.1 (7.8)	2.3 (2.8)	3.0 (3.7)	−0.7 (−1.5, 0.0)	0.06
Median (IQR)	5.0 (2.3, 8.4)	3.1 (1.3, 9.2)	1.4 (0.5, 2.8)	1.4 (0.8, 4.3)		
Secondary outcomes						
Number of urgency episodes per week						
Mean (SD)	5.1 (3.4)	9.6 (9.4)	3.2 (2.5)	2.7 (2.6)	0.5 (−0.4, 1.4)	0.23
Median (IQR)	5.5 (2.8, 6.8)	7.0 (4.1, 11.3)	3.1 (1.1, 4.5)	1.6 (0.8, 3.8)		
Number of urge episodes per week						
Mean (SD)	2.3 (2.0)	3.1 (3.7)	0.6 (0.8)	0.9 (1.4)	−0.3 (−0.8, 0.2)	0.27
Median (IQR)	2.0 (1.0, 2.8)	1.9 (0.9, 3.3)	0.3 (0.0, 0.9)	0.5 (0.1, 1.0)		
Number of passive FI episodes per week						
Mean (SD)	4.3 (6.5)	4.1 (5.0)	1.7 (2.8)	2.1 (3.8)	−0.5 (−1.3, 0.4)	0.28
Median (IQR)	2.9 (0.0, 5.6)	1.8 (0.0, 7.1)	0.8 (0.0, 1.4)	0.4 (0.0, 1.6)		
Number of episodes of leakage of flatus per week						
Mean (SD)	9.5 (10.0)	39.3 (41.4)	15.0 (22.4)	24.7 (38.2)	−9.6 (−20.9, 1.6)	0.09
Median (IQR)	8.3 (0.5, 16.5)	22.9 (8.1, 66.6)	4.8 (1.6, 19.1)	5.9 (1.4, 31.9)		
% of days patient used loperamide (%)						
Mean (SD)	49.8 (38.6)	35.4 (41.6)	30.0 (37.3)	32.7 (43.2)	−2.7 (−8.6, 3.2)	0.34
Median (IQR)	56.5 (22.2, 75.0)	9.1 (0.0, 71.4)	9.1 (1.8, 64.3)	7.3 (0.0, 83.9)		
% of days faecal incontinence limited a patient's social activities (%)						
Mean (SD)	32.9 (41.2)	61.2 (39.8)	15.6 (25.9)	18.7 (33.6)	−3.1 (−19.4, 13.3)	0.69
Median (IQR)	12.5 (0.0, 56.5)	76.4 (18.5, 96.4)	0.0 (0.0, 20.1)	3.7 (0.0, 15.4)		

E‐event linked recordings	*N* = 8	*N* = 6	N = 7	*N* = 7	Mean difference^c^ (95% CI)	*p* ^d^
Number of episodes of faecal material per week						
Mean (SD)	4.2 (3.7)	2.8 (2.6)	0.8 (1.0)	2.2 (2.0)	−1.5 (−3.5, 0.5)	0.12
Median (IQR)	2.5 (1.6, 7.9)	1.9 (0.8, 4.3)	0.5 (0.0, 1.3)	2.3 (0.0, 2.8)		
Number of episodes of leakage of flatus per week						
Mean (SD)	11.5 (12.5)	9.0 (11.4)	8.8 (6.5)	10.3 (11.7)	−1.5 (−12.0, 8.9)	0.73
Median (IQR)	9.9 (0.1, 18.8)	4.8 (2.3, 11.5)	9.0 (2.0, 15.8)	6.5 (3.5, 11.8)		
Number of episodes of urgency without incontinence per week						
Mean (SD)	5.8 (2.4)	12.3 (13.3)	1.8 (1.7)	1.4 (1.0)	0.4 (−1.1, 1.9)	0.51
Median (IQR)	5.9 (3.9, 8.0)	6.5 (4.3, 20.8)	1.0 (0.8, 4.0)	1.0 (0.8, 2.0)		

^a^ Baseline data are missing for four SNM/SHAM participants and two SHAM/SNM participants (see Figure 1).

^b^ Crossover (T28–T32) data missing for eight SNM/SHAM participants and 15 SHAM/SNM participants (see Figure 1).

^c^ Direction of difference: SNM–SHAM; hence negative differences indicate fewer episodes with SNM versus SHAM.

^d^ Two‐sided *p*‐value from paired *t*‐test SNM versus SHAM.

### Implantation details and intraoperative data

Implantation details and intra‐operative responses are shown in Tables S4 and S5. Test stimulation was performed using a tined lead in 67.6% of patients and the lead was positioned in the S3 foramen in most patients (91.4%). Mean operating time was 42 min (SD 21). Implantation was reported as successful in all cases. Motor responses were used in most cases to guide lead placement. Bellows contraction was observed in most implantations (93.5%), followed by big toe flexion (80.0%) and anal sphincter contraction (62.5%). Motor or sensory responses <1 V for ≥3 electrodes were achieved in 50% of cases. Post‐operative programming data are shown in Table S6.

### Primary outcome

**FIGURE 2:**
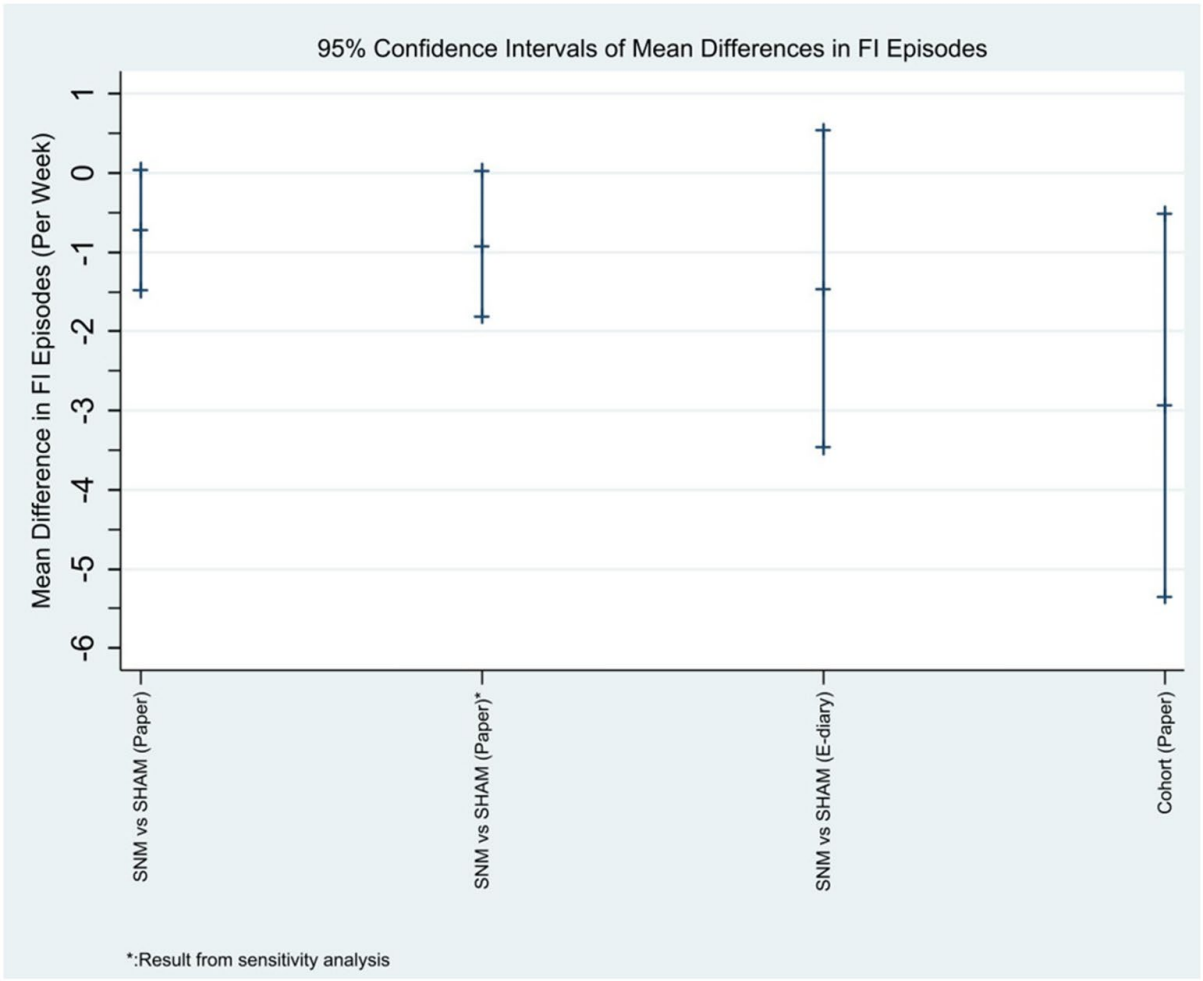
Comparison of effect estimates for the primary outcome (FI episodes per week) based on different methods of detection and for the sensitivity analysis.

The effect of SNM versus SHAM on the number of FI episodes per week is shown in Table 2 and Figure 2 for the 16 patients with complete data. SNM led to a non‐significant mean reduction of 0.7 FI episodes per week compared to SHAM (CI −1.5 to 0.0; *p* = 0.06). Table S8 details the number of days the paper bowel diaries were completed for the primary outcome by the randomised patients throughout the study. A sensitivity analysis (best‐case scenario in which missing data were imputed as zero FI episodes when at least one other item of the bowel diary had been completed for that day) resulted in a slightly greater effect size (FI episodes −0.9; CI −1.8 to 0.0; *p* = 0.04; Table S9; Figure 2).

### Secondary outcomes

In the small number of patients (*N* = 7/14) who had complete data from the e‐event recorder (as an alternative measurement of the primary outcome), the effect size of SNM versus SHAM was greater but less precise (FI episodes −1.5; CI −3.5 to 0.5; *p* = 0.12; Table 2; Figure 2). Bland–Altman analysis showed that paper bowel diaries recorded 7.2 more FI episodes over the 28‐day period than the e‐event recordings (Figure S3). SNM (vs. SHAM) led to a non‐significant reduction in all other FI symptoms (except for faecal urgency; Table 2).

The results from the secondary outcomes are summarised in Table S3, with most showing small non‐significant directional changes in favour of SNM. The difference in overactive bladder symptoms (OAB‐Q) was most marked, although not statistically significant (adj. mean difference −10.80; CI −23.02 to 1.14; *p* = 0.08). The difference in satisfaction score (Likert scale) between arms was small, with no indication that patients felt more satisfied with SNM than SHAM.

### Patient perception of group allocation

Perception of group allocation is shown in Table S10. The available data demonstrate that blinding was effective, that is, patient's perception of receiving SNM was equally spread throughout all four periods.

### Comparison of crossover effect sizes with baseline

Although baseline data have no relevance to detecting changes in the crossover phase, the large differences in the number of FI episodes observed between baseline and results in both arms (regardless of period) warrant mention. Mean FI episodes per week at baseline were 6.4 (SD 6.2) in those patients with complete crossover data, compared to a mean of 3.0 (SD 3.7) for SHAM during crossover. This difference is greater than the within‐crossover effect of SNM versus SHAM (FI episodes −0.7; CI −1.5 to 0.0). Similar results were seen for all other outcomes (e.g. for St Mark's incontinence score, almost no within‐crossover effect [−0.15 points] but a reduction of approximately 5 points [19–14] from baseline for SHAM).

### Cohort follow‐up

Table 3 details the bowel diary outcomes in the 19 participants with complete data at baseline and at the end of the study (58 weeks follow‐up). Compared to baseline, symptom improvement was observed for all recorded outcomes. The number of FI episodes reduced to a mean of 3.2 (SD 3.3) episodes per week (vs. baseline: 6.2; SD 5.9). In contrast to the crossover phase (non‐significant increase of urgency episodes), the mean number of urgency episodes per week decreased from 7.7 (SD 6.0) at baseline to 2.8 (SD 3.2) at 58 weeks' follow‐up. Mean loperamide usage decreased from 47.0% of days at baseline to 32.0% of days at follow‐up. The percentage of days FI limited patients' social activities also reduced from 43.4% at baseline to 25.3% at follow‐up.

**TABLE 3:** Paper bowel diary outcomes at baseline and end of study (T58) for participants with data available at both timepoints.

Outcomes	Baseline	End of study (T58)
Primary outcome	*N* = 19	*N* = 19
Number of FI episodes per week		
Mean (SD)	6.2 (5.9)	3.2 (3.3)
Median (IQR)	4.5 (2.3, 9.3)	2.5 (0.0, 5.3)

Secondary outcomes		
Other paper bowel diary measures	*N* = 19	*N* = 19
Number of urgency episodes per week		
Mean (SD)	7.7 (6.0)	2.8 (3.2)
Median (IQR)	6.5 (3.8, 10.5)	2.0 (0.5, 3.8)
Number of urge incontinence episodes per week		
Mean (SD)	2.2 (1.7)	1.4 (2.1)
Median (IQR)	2.0 (1.0, 2.8)	0.3 (0.0, 2.2)
Number of passive faecal incontinence episodes per week		
Mean (SD)	4.0 (6.0)	1.8 (2.8)
Median (IQR)	1.5 (0.0, 5.6)	0.5 (0.0, 4.0)
Number of episodes of leakage of flatus per week		
Mean (SD)	28.2 (37.5)	15.3 (20.8)
Median (IQR)	13.3 (5.3, 37.3)	3.9 (0.8, 32.3)
% of days patient used loperamide for their incontinence symptoms (%)		
Mean (SD)	47.0 (35.6)	32.0 (42.0)
Median (IQR)	56.5 (11.1, 74.1)	7.1 (0.0, 78.6)
% of days faecal incontinence limited a patient's social activities (%)		
Mean (SD)	43.4 (37.9)	25.3 (41.5)
Median (IQR)	25.9 (10.7, 77.8)	0.0 (0.0, 61.5)

Improvement was also observed for the other secondary outcomes at 58 weeks' follow‐up (Table S11). The mean St Mark's incontinence score decreased from 19.0 (SD 2.3) to 13.5 (SD 5.7). FI‐QOL improved on all domains, as did the SF‐ICIQ‐B and EQ‐5D‐5L. Patient‐reported treatment satisfaction at 58 weeks' follow‐up (recorded on a 0–100 Likert scale) was high (mean 73.8; SD 20.8).

### Adverse events (crossover and cohort follow‐up)

A total of 10 adverse events occurred during the study (Table S12). One event was classified as a serious adverse device event (SADE) although it did not require overnight hospitalisation.

## DISCUSSION

The SUBSoNIC trial terminated early and failed to recruit and retain sufficient patient numbers. The COVID‐19 pandemic was the main reason for these failures, resulting in the inability to continue face‐to‐face patient visits, the redeployment of research staff to COVID‐19‐facing clinical roles and the cancelling of SNM surgery due to lack of priority. These reasons caused disruption of contact between the study team and patients, resulting in losses to follow‐up and incomplete data collection. There were also other causes that are common to many complex trials in the NHS, including the slow opening of participating sites resulting from contractual and governance issues. Accepting the caveats of significant under‐recruitment (39/90 patients randomised) and attrition (primary outcome available in 16 patients), the SUBSoNIC trial is the first randomised controlled trial of SNM in a treatment‐naïve population with adequate washout periods, and probably the first with effective double blinding.

Due to significant under‐recruitment, the study findings should be interpreted with caution from the perspective of statistical and clinical inference. With data on the primary outcome available in only 16 patients, it is impossible to predict the effect in a hypothetical situation where the full sample size would have been recruited. However, the available data show that SNM may have a biological effect over and above SHAM. The mean difference in FI episodes (−0.7 FI episodes per week; CI −1.5 to 0.0) between SNM (2.3 FI episodes per week; SD 2.8) and SHAM (3.0 FI episodes per week; SD 3.7) equals, at the upper confidence limit of 1.5, a mean reduction of 50%. Thus, in comparison with the reduction of 30% assumed in the sample size calculation, our results are inconclusive but do not rule out clinically important effects. The result was certainly affected by missing data on the bowel diaries, even in those patients who provided data in both intervention periods. Despite clear instructions to fill in a zero for those days where no FI episodes occurred, several bowel diaries were not entirely completed (data shown in Table S8). Whether this reflects absence of symptoms or a day of failed compliance remains uncertain. Sensitivity analysis imputing missing data for zero episodes of FI resulted in a slight increase of effect size (FI episodes −0.9; CI −1.8 to 0.0; *p* = 0.04).

The lack of any ideal FI outcome measure is well acknowledged [18, 23]. Despite the success of e‐recording technology in other disciplines [24, 25], and despite Patient and Public Involvement (PPI) in the development of the e‐recording device, compliance was even less than for paper bowel diaries. It is unknown whether the use of such a method of recording in isolation would improve compliance (i.e. participants perhaps did not want to do both). Overall, the method of obtaining diary data and its interpretation confers significant changes in inference that confirm the frailties of such approaches for future studies.

When comparing the findings of SUBSoNIC with previous studies, it should be noted that the evidence base from randomised controlled trials is poor. A Cochrane review included only six randomised trials (four crossover trials and two parallel group randomised controlled trials) [9]. Of the crossover trials, one included two patients only [26], and the other was published only as an abstract reporting data in 7 patients only [27]. Of the other two crossover studies, the highly cited study by Leroi et al. remains the pivotal trial in providing evidence for clinical efficacy of SNM [28]. Of the 34 preselected patients, 27 were included in the crossover phase (1 month active stimulation/SHAM stimulation, washout of only 1 week), and 24 completed the study (10 patients were excluded due to adverse events, lack of efficacy or protocol violations). Although most patients favoured active stimulation over SHAM, the study failed to show a clinically relevant reduction in FI episodes. It should be acknowledged that Leroi et al. did not specifically study subsensory stimulation (which questions adequate blinding of patients) and no data were provided as to whether blinding was successful. Further, active stimulation was provided for a period of 1–3 months prior to crossover, with inclusion of prior ‘successful’ patients only. Although the crossover design of the SUBSoNIC trial did not permit full adherence to an intention‐to‐treat principle (i.e. from the start of trial therapy with test stimulation), all newly implanted patients were randomised, rather than patients who have already been selected based on successful chronic therapy. Differences aside, the overall effect of SNM versus SHAM (mean reduction of 1 FI episode per week) reported by Leroi et al. was comparable with the results of the SUBSoNIC trial. Further, as in the current trial, changes to faecal urgency and summative symptom scores failed to achieve significance between intervention periods.

An important finding from the SUBSoNIC study is that the effect of SNM (independent of how it was measured) was much smaller than a possible placebo effect. Mean FI episodes per week was approximately 7 at baseline, and 3 (SD 3.7) in the SHAM period. The reduction in FI episodes was also observed at 58 weeks' follow‐up, where FI episodes reduced from 6.2 (SD 5.9) at baseline to 3.2 (SD 3.3). This possible placebo effect concurs with other neuromodulation studies [29] and double‐blind trials for other treatments in patients with FI [10]. Although the effect of subsensory stimulation is only investigated in a few studies [30], we do not believe that stimulation at a maximum of 0.05 V in the SHAM group contributed to symptom reduction. As per FDA recommendation [31], data were collected on the adequacy of the blinding and demonstrated the success of our approach. The physiological basis of the placebo effect is well understood for certain conditions, and SHAM interventions generally produce greater placebo effects than pharmacological interventions in clinical trials [32]. Understanding the genuine versus placebo effect of SNM is relevant to reduce cost utilisation as well as to our fundamental understanding of the pathophysiology of FI. However, COVID‐19 could also have influenced this finding. Some patients completed the baseline questionnaires before the pandemic, and it is possible that FI episodes occurred less frequently (in both arms) as patients had to stay at home (close to the toilet) during lockdown.

All secondary outcomes favoured SNM with small non‐significant effects. Compared to the other outcomes, the effect was slightly greater measured with the ICIQ‐OAB (non‐significant reduction of −10.8; CI −23.0 to 1.14), which evaluates overactive bladder and related impact on quality of life [21]. Urinary symptoms often co‐exist with FI [33], and SNM is considered a main therapy for overactive bladder symptoms [34, 35]. In contrast to all other outcomes, faecal urgency increased during SNM, which may be explained by a genuine treatment effect whereby episodes of urgency that previously resulted in incontinence have been ‘reduced’ to urgency without incontinence because of increased perception. This is in keeping with extensive observational data that report improvements of deferment time with SNM [36] and the normalisation of rectal sensory function [37, 38].

In conclusion, the SUBSoNIC clinical trial failed to find conclusive evidence of the experimental efficacy of SNM. However, we believe that, in the absence of a pandemic, and with lessons learned from the SUBSoNIC trial (mainly on capturing bowel diary data), the trial could be repeated and delivered successfully. Further demonstration of the experimental efficacy of SNM for FI remains important as SNM is a high‐cost and invasive therapy. Bowel diary approaches require considerable scrutiny if used as outcome measures in the future.

## AUTHOR CONTRIBUTIONS

**Paul F. Vollebregt:** Writing – original draft; project administration; data curation. **Yan Li Goh:** Data curation; project administration; writing – review and editing. **Anil Bagul:** Writing – review and editing; project administration; data curation. **Claire Chan:** Formal analysis; methodology; writing – review and editing. **Tom Dudding:** Conceptualization; funding acquisition; methodology; writing – review and editing. **Paul Furlong:** Conceptualization; funding acquisition; methodology; writing – review and editing. **Shaheen Hamdy:** Conceptualization; funding acquisition; methodology; writing – review and editing. **Joanne Haviland:** Methodology; writing – review and editing; formal analysis. **Richard Hooper:** Conceptualization; methodology; writing – review and editing; funding acquisition. **James Jones:** Conceptualization; funding acquisition; methodology; writing – review and editing. **Eleanor McAlees:** Data curation; project administration; writing – review and editing. **Christine Norton:** Conceptualization; funding acquisition; methodology; writing – review and editing. **P. Ronan O'Connell:** Conceptualization; funding acquisition; writing – review and editing; methodology. **Michael Powar:** Data curation; project administration; writing – review and editing. **S. Mark Scott:** Conceptualization; funding acquisition; methodology; writing – review and editing. **Natasha Stevens:** Project administration; writing – review and editing. **Kerry Tubby:** Writing – review and editing; project administration. **Sian Worthen:** Writing – review and editing. **Yuk Lam Wong:** Formal analysis; writing – review and editing. **Charles H. Knowles:** Conceptualization; funding acquisition; methodology; writing – review and editing; supervision; writing – original draft.

## FUNDING INFORMATION

This project was funded by the National Institute for Health and Care Research (NIHR) EME programme and by Medtronic Inc.

## CONFLICT OF INTEREST STATEMENT

Shaheen Hamdy holds stocks/shares in the company Anisys Ltd., which is developing a probe to assess anorectal physiology and holds stocks/shares, and is CSO of Phagenesis Ltd., a company focusing on treatments for dysphagia. Christine Norton has received Speakers fees from Janssen, WebMD, Medscape, Merck Pharmaceuticals, Tillotts Pharma UK and funding for the advisory board of Pfizer. S Mark Scott has received honoraria from Laborie for teaching (Webinars and hands‐on training courses). Charles H Knowles is a consultant and speaker for Medtronic Inc.; he is a founder, inventor, shareholder and Chief Medical Officer of Amber Therapeutics Ltd., a company developing a new implanted pudendal nerve stimulation system for urinary incontinence. Other authors have no conflicts of interest.

## ETHICS STATEMENT

Ethical approval was received from the London City and East Research Ethics Committee (Reference 17/LO/1060, IRAS 187783). The trial is registered on an open access registry: ISRCTN98760715.

## PATIENT CONSENT STATEMENT

All participants provided informed consent.

## COLLABORATORS

Karen Telford (Department of Colorectal Surgery, Manchester University NHS Foundation Trust, Manchester, UK), Wesley Lai (University Hospitals Plymouth NHS Trust, Plymouth, UK), Carmen Ho (University Hospitals of Leicester NHS Trust, Leicester, UK), Ann Hanly (St Vincent's University Hospital, Dublin, Ireland).

## Supporting information


Figure S1.

## Data Availability

The data that support the findings of this study are available from the corresponding author upon reasonable request.
