# Supplementary material for: Clinical efficacy of subsensory sacral neuromodulation in adults with faecal incontinence: The SUBSoNIC crossover randomised controlled trial and cohort follow‐up study
Source: Colorectal Dis. 2025 Nov 11;27(11):e70308. doi: 10.1111/codi.70308 (PMC12605715; doi:10.1111/codi.70308)
Supplement: Supplementary file 1 — Figure S1. [file CODI-27-0-s001.docx]

**Supplementary Tables and Figures**

***Supplementary Figure 1.*** *Example photograph of touchscreen icons on e-recording device.*


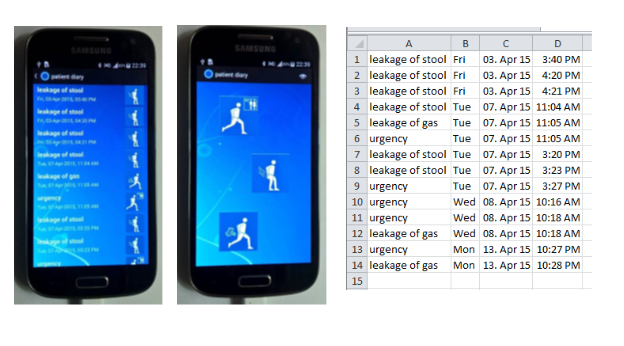


***Supplementary Figure 2.*** *Number of patients who declined study participation and reasons for ineligibility.*

***Supplementary Table 1.*** *Gynecological and obstetric history at baseline.*

|  | **Randomised allocation** | |
| --- | --- | --- |
|  | **SNM/SHAM**  **N=16*** | **SHAM/SNM**  **N=20*** |
| **>1yr post-menopausal (%)**  No  Yes  Missing | 6 (38)  10 (63)  0 | 3 (18)  14 (82)  3 |
| **Sterilisation (%)**  No  Yes  Missing | 10 (63)  6 (38)  0 | 9 (53)  8 (47)  3 |
| **Past obstetric history (%)**  No  Yes  Missing | 0 (0)  16 (100)  0 | 1 (6)  17 (94)  2 |
| **Number of vaginal deliveries**  Mean (SD)  Median (IQR) | N=16  1.7 (1.1)  2.0 (1.0, 2.5) | N=17  2.7 (1.5)  2.0 (2.0, 3.0) |
| **Number of cesareans**  Mean (SD)  Median (IQR) | N=16  0.4 (0.7)  0.0 (0.0, 0.5) | N=17  0.0 (0.0)  0.0 (0.0, 0.0) |
| **Number of forceps/ventouse**  Mean (SD)  Median (IQR) | N=16  0.8 (0.8)  0.0 (1.0, 1.0) | N=17  0.4 (0.6)  0.0 (0.0, 1.0) |
| **Number of episiotomies**  Mean (SD)  Median (IQR) | N=16  0.8 (0.8)  1.0 (0.0, 1.0) | N=17  1.1 (1.4)  1.0 (0.0, 1.0) |
| **Past obstetric tears**  Yes  No | N=16  11 (69)  5 (31) | N=17  13 (76)  4 (24) |
| **Number of obstetric tears**  Mean (SD)  Median (IQR) | N=16  0.8 (0.6)  1.0 (0.0, 1.0) | N=17  1.3 (1.4)  1.0 (1.0, 2.0) |
| **Degree of worst tear – no. (%)**  1  2  3a  3b  3c  3d  Missing | N=11  0 (0)  0 (0)  3 (50)  0 (0)  1 (17)  2 (33)  5 | N=13  0 (0)  1 (14)  2 (29)  0 (0)  1 (14)  3 (43)  6 |

*Footnote:*

** Percentages calculated excluding missing data.*

***Supplementary Table 2.*** *Clinical examination at baseline.*

|  | **Randomised allocation** | |
| --- | --- | --- |
|  | **SNM/SHAM**  **N=17*** | **SHAM/SNM**  **N=22*** |
| **Gross neurology (%)**  Normal  Poor  Wheelchair  Other  Missing | 17 (100)  0 (0)  0 (0)  0 (0)  0 | 21 (95)  0 (0)  0 (0)  1^2^ (5)  0 |
| **Abdominal exam (%)**  Normal  Scars  Distension  Organomegaly  Palpable faecal loading  Missing | 11 (69)  5 (31)  0 (0)  0 (0)  0 (0)  1 | 16 (76)  5 (24)  0 (0)  0 (0)  0 (0)  1 |
| **Perineal rectal exam (%)**  Normal  Visible soiling  Excoriation | 15 (88)  2 (12)  0 | 18 (82)  3 (14)  1 (5) |
| **(If yes to visible soiling) Type of soiling (%)**  Staining  Frank  Missing | N=2  1 (100)  0 (0)  1 | N=3  2 (100)  0 (0)  1 |
| **Scars (%)**  None  Tear  Episiotomy  Hemorrhoidectomy  Fistulotomy  Other  Missing | 10 (59)  3 (18)  3 (18)  0 (0)  0 (0)  1 (6)  0 | 13 (59)  3 (14)  0 (0)  0 (0)  0 (0)  6 (27)  0 |
| **Perineal position (%)**  Normal  Descent at rest  Descent at straining >2cm  Complete effacement on straining  Ballooning perineum  Missing | 14 (88)  2 (13)  0 (0)  0 (0)  0 (0)  1 | 17 (77)  4 (18)  1 (5)  0 (0)  0 (0)  0 |
| **External prolapse (%)**  None  Mucosal  Full thickness  Missing | 17 (100)  0 (0)  0 (0)  0 | 22 (100)  0 (0)  0 (0)  0 |
| **Internal prolapse (%)**  None  Intussusception  Missing | 17 (100)  0 (0)  0 | 21 (95)  1 (5)  0 |
| **Anal closure (%)**  Normal  Open/gaping  Anal sphincter defect  Anterior  Other  Missing | 11 (64)  4 (24)  1 (6)  1 (6)  0 (0)  0 | 16 (76)  3 (14)  1 (5)  1 (5)  0 (0)  1 |
| **Anal tone (%)**  Normal  Reduced  Missing | 2 (12)  15 (88)  0 | 11 (50)  11 (50)  0 |
| **Anal squeeze (%)**  Normal  Reduced  Reduced and non-concentric  Missing | 2 (12)  14 (82)  1 (6)  0 | 3 (14)  18 (82)  1 (5)  0 |
| **Other anal findings (%)**  None  Hemorrhoids  Fissure  Other  Missing | 15 (88)  1 (6)  0 (0)  1 (6)  0 | 15 (71)  4 (19)  0 (0)  2 (10)  1 |
| **(If yes to hemorrhoids) Grade – no. (%)**  1  2  3  4  Missing | N=1  1 (100)  0 (0)  0 (0)  0 (0)  0 | N=4  0 (0)  0 (0)  0 (0)  0 (0)  4 |
| **Faecal loading (%)**  None  Soft  Hard  Impacted  Missing | 16 (94)  1 (6)  0 (0)  0 (0)  0 | 19 (86)  3 (14)  0 (0)  0 (0)  0 |
| **Rectocoele (%)**  None  Small  Moderate  Large  Missing | 12 (71)  5 (29)  0 (0)  0 (0)  0 | 17 (77)  4 (18)  1 (5)  0 (0)  0 |
| **Pelvic floor movement on straining (%)**  Appropriate relaxation  No relaxation  Contraction  Missing | 14 (88)  0 (0)  2 (13)  1 | 17 (81)  2 (10)  2 (10)  1 |

*Footnote:*

** Percentages calculated excluding missing data.*

***Supplementary Table 3.*** *Symptom questionnaires at baseline and during crossover.*

|  | **Baseline** | | **Crossover** | | | |
| --- | --- | --- | --- | --- | --- | --- |
|  | **SNM/SHAM** | **SHAM/SNM** | **SNM** | **SHAM** | **Mixed linear regression analysis** | |
|  |  |  |  |  | **Adjusted mean difference***  **(95% CI)** | **p-value** |
| **St Mark’s incontinence score^1^** | | | | | | |
| Original St Mark’s score  Mean (SD)  Median (IQR) | N=17  18.2 (2.6)  19.0 (16.0, 20.0) | N=22  19.1 (1.9)  19.0 (18.0, 20.0) | N=21  13.9 (4.9)  14.0 (11.0, 18.0) | N=22  14.2 (5.2)  16.5 (12.0, 18.0) | -0.15 (-2.11, 1.81) | 0.88 |
| Modified St Mark’s score  Mean (SD)  Median (IQR) | N=17  17.8 (2.9)  19.0 (16.0, 20.0) | N=22  18.5 (2.6)  19.0 (17.0, 20.0) | N=21  13.0 (5.7)  14.0 (7.0, 16.0) | N=23  13.4 (5.6)  15.0 (11.0, 18.0) | 0.11 (-2.17, 2.39) | 0.93 |
| **Assessment of OverActive Bladder symptoms (OAB-Q) short form** | | | | | | |
| OAB-Q SF score^2^  Mean (SD)  Median (IQR) | N=17  24.3 (23.0)  13.3 (13.3, 26.7) | N=22  42.4 (27.1)  38.3 (23.3, 60.0) | N=21  13.9 (4.9)  14.0 (11.0, 18.0) | N=22  14.2 (5.2)  16.5 (12.0, 18.0) | -10.80  (-23.02, 1.14) | 0.08 |
| **Fecal Incontinence Quality of Life (FI QOL)^3^** | | | | | | |
| Lifestyle mean score  Mean (SD)  Median (IQR) | N=17  2.0 (0.7)  1.8 (1.6, 2.1) | N=21  2.0 (0.7)  1.8 (1.6, 2.5) | N=20  2.8 (0.9)  2.9 (1.9, 3.5) | N=23  2.7 (0.9)  2.7 (1.9, 3.6) | -0.11 (-0.38, 0.16) | 0.42 |
| Coping behaviour mean score  Mean (SD)  Median (IQR) | N=13  1.4 (0.6)  1.3 (1.0, 1.4) | N=19  1.4 (0.5)  1.2 (1.1, 1.3) | N=19  2.1 (0.6)  2.2 (1.7, 2.6) | N=24  1.9 (0.7)  1.8 (1.3, 2.7) | 0.06 (-0.24, 0.36) | 0.71 |
| Depression/self-perception mean score  Mean (SD)  Median (IQR) | N=13  2.1 (0.5)  2.0 (1.6, 2.5) | N=17  2.0 (0.4)  1.9 (1.8, 2.2) | N=20  2.6 (0.7)  2.5 (2.2, 3.1) | N=23  2.5 (0.8)  2.3 (1.8, 3.5) | -0.01 (-0.26, 0.24) | 0.96 |
| Embarrassment mean score  Mean (SD)  Median (IQR) | N=17  1.8 (1.0)  1.3 (1.0, 2.0) | N=21  1.5 (0.5)  1.3 (1.3, 1.7) | N=22  2.2 (0.8)  2.2 (1.7, 2.7) | N=24  2.1 (0.8)  2.0 (1.7, 2.5) | -0.05 (-0.29, 0.19) | 0.68 |
| **Short form International Consultation on Incontinence Bowel Questionnaire (SF-ICIQ-B)** | | | | | | |
| SF-ICIQ-B mean score^4^  Mean (SD)  Median (IQR) | N=17  9.3 (0.9)  9.9 (8.5, 10.0) | N=22  9.2 (1.1)  9.6 (8.8, 10.0) | N=22  7.7 (1.7)  8.0 (6.6, 9.0) | N=24  8.5 (1.4)  9.1 (7.4, 9.8) | -0.65 (-1.46, 0.17) | 0.12 |
| **EuroQol Health Outcome Measure (EQ-5D-5L)** | | | | | | |
| Summary index value^5^  Mean (SD)  Median (IQR) | N=17  0.71 (0.21)  0.78 (0.64, 0.85) | N=21  0.79 (0.16)  0.83 (0.75, 0.90) | N=22  0.71 (0.26)  0.77 (0.52, 0.92) | N=24  0.76 (0.20)  0.81 (0.70, 0.90) | -0.02 (-0.10, 0.05) | 0.58 |
| EQ-VAS score^6^  Mean (SD)  Median (IQR) | N=17  66.7 (19.3)  70.0 (50.0, 80.0) | N=21  70.2 (14.8)  70.0 (60.0, 80.0) | N=22  71.5 (17.7)  75.0 (60.0) | N=24  71.0 (18.3)  75.0 (60.0, 85.0) | 1.58 (-3.72, 6.88) | 0.56 |
| **Likert scale of patient’s global impression of treatment success** | | | | | | |
| Satisfaction score^7^  Mean (SD)  Median (IQR) |  |  | N=22  61.6 (28.2)  67.5 (50.0, 80.0) | N=24  60.2 (31.7)  77.5 (30.0, 80.0) | -3.22 (-18.53, 12.10) | 0.68 |

*Footnote: ** *Difference in means adjusted for period and sex (fixed effects) and random effects for centre and participant. Direction of difference: SNM-sham; hence negative differences indicate lower scores with SNM versus sham. ^1^ For both the modified and original St Mark’s scores, the minimum score is 0 and the maximum total score is 24; high scores indicate more incontinence. ^2^ The OAB-Q short form score has a range of 0-100; higher scores indicate greater symptom bother. ^3^ Each FI QOL subscale score ranges from 1 to 4; higher scores indicate greater quality of life.^4^ SF-ICIQ-B mean score has a range of 0-10; higher scores indicate greater importance of bowel incontinence on life issues. ^5^ Summary index values will be presented against country specific value sets. ^6^ VAS records the respond’s self-rated health on a scale from 0 to 100; higher scores indicate impressions of better health. ^7^ Satisfaction score indicates the extent to which the patient feels that their symptoms have improved compared with before the study. The score ranges from 0%=not at all to 100%=complete cure.*

***Supplementary Table 4.*** *Implantation details.*

| **Lead details** | **Summary measure (%)**  **N=39*** |
| --- | --- |
| **Tined lead already implanted (%)**  Yes  No | N=37  25 (68)  12 (32) |
| **Lead type (%)**  3889  3093 | N=34  34 (100)  0 |
| **Curved stylet used (%)**  Yes  No | N=37  36 (97)  1 (3) |
| **Side implanted (%)**  Left  Right | N=37  22 (60)  15 (41) |
| **Radiological Foramen (%)**  S3  S4  S2 | N=37  34 (92)  3 (8)  0 |
| **Dominant Electrode (%)**  3  2  1  0 | N=35  9 (26)  8 (23)  13 (37)  5 (14) |
| **Number of Electrodes within sacral foramina (%)**  ≥3  2  1  0 | N=37  24 (65)  0  13 (35)  0 |
| **Type of anaesthesia (%)**  General  Local  Local with sedation | N=36  26 (72)  0  10 (28) |
| **(If General) Time in mins**  Mean (SD)  Median (IQR)  **Paralysis used (%)**  Yes  No  **If paralysis used, paralysis reversed (%)**  Yes  No | N=22  70.6 (27.9)  60.0 (50.0, 95.0)  N=24  6 (25)  18 (75)  N=5  0  5 (100) |
| **(If Local with sedation) Type (Multi-select) (%)**  Lidocaine/Xylocaine  Bupivicaine  Propofol  Midazolam  Fentanyl  Other | N=10  3 (30)  5 (50)  4 (40)  4 (40)  2 (20)  4 (40) |
| **Prophylactic antibiotics used (%)**  Yes  No | N=36  35 (97)  1 (3) |
| **Estimated blood loss <10 mls (%)**  <10 mls  Other | N=37  35 (95)  2 (5) |
| **Duration of surgery knife to skin (mins)**  Mean (SD)  Median (IQR) | N=34  41.9 (21.4)  35.0 (29.0, 55.0) |
| **Implantation of permanent device successfully completed (%)**  Yes  No | N=37  37 (100)  0 |
| **Post-operative stay (hours)**  Mean (SD)  Median (IQR) | N=26  3.9 (4.9)  3.0 (2.0, 4.0) |

*Footnote: * Denominators may vary for individual measures due to missing data.*

*Supplementary Table 5. Intra-operative responses.*

|  | **Summary measure (%)**  **N*=39** |
| --- | --- |
| **Intra-operative motor responses**** | **N=34** |
| **Ideal response**  All 4 electrodes < 1 Volt, bellows contraction, and big toe flexion | N=33  6 (18) |
| **Motor threshold**  4 electrodes < 1 Volt  3 electrodes < 1 Volt  2 electrodes < 1 Volt  1 electrode < 1 Volt  0 electrode < 1 Volt | N=33  10 (30)  6 (18)  7 (21)  6 (18)  4 (12) |
| **Pelvic floor response – bellows contraction**  4 electrodes  3 electrodes  2 electrodes  1 electrode  0 electrode | N=31  26 (84)  2 (7)  1 (3)  0  2 (7) |
| **Pelvic floor response – anal sphincter contraction**  4 electrodes  3 electrodes  2 electrodes  1 electrode  0 electrode | N=24  12 (50)  2 (8)  1 (4)  0  9 (38) |
| **Foot response – big toe flexion**  4 electrodes  3 electrodes  2 electrodes  1 electrode  0 electrode | N=30  18 (60)  3 (10)  2 (7)  1 (3)  6 (20) |
| **Foot response – forefoot flexion**  4 electrodes  3 electrodes  2 electrodes  1 electrode  0 electrode | N=19  0  1 (5)  0  2 (11)  16 (84) |
| **Intra-operative sensory responses**** | **N=3** |
| **Ideal response**  All 4 electrodes < 1 Volt, and anus sensation | N=3  0 |
| **Sensory threshold**  4 electrodes < 1 Volt  3 electrodes < 1 Volt  2 electrodes < 1 Volt  1 electrode < 1 Volt  0 electrode < 1 Volt | N=3  1 (33)  1 (33)  0  0  1 (33) |
| **Anus sensation**  4 electrodes  3 electrodes  2 electrodes  1 electrode  0 electrode | N=1  1 (100)  0  0  0  0 |
| **Perineum sensation**  4 electrodes  3 electrodes  2 electrodes  1 electrode  0 electrode | N=1  1 (100)  0  0  0  0 |
| **Other sensation**  4 electrodes  3 electrodes  2 electrodes  1 electrode  0 electrode | NA |

*Footnote: * Denominators may vary for individual measures due to missing data. **Note that patients either complete intra-operative motor responses or intra-operative sensory responses. Moreover, intra-operative sensory thresholds are only relevant to patients who have had local anesthetic.*

***Supplementary Table 6.*** *Initial programming data.*

|  | **Summary measure (%)**  **N*=39** |
| --- | --- |
| **Monopolar evaluation details** |  |
| **Threshold**  4 electrodes < 1 Volt  3 electrodes < 1 Volt  2 electrodes < 1 Volt  1 electrode < 1 Volt  0 electrode < 1 Volt | N=34  18 (53)  8 (24)  5 (15)  2 (6)  1 (3) |
| **Anus sensation**  4 electrodes  3 electrodes  2 electrodes  1 electrode  0 electrode | N=34  4 (12)  5 (15)  4 (12)  4 (12)  17 (50) |
| **Perineum sensation**  4 electrodes  3 electrodes  2 electrodes  1 electrode  0 electrode | N=34  2 (6)  1 (3)  3 (9)  4 (12)  24 (71) |
| **Genital sensation**  4 electrodes  3 electrodes  2 electrodes  1 electrode  0 electrode | N=34  0  1 (3)  1 (3)  7 (21)  25 (74) |
| **Other sensation**  4 electrodes  3 electrodes  2 electrodes  1 electrode  0 electrode | N=34  12 (35)  5 (15)  4 (12)  8 (24)  5 (15) |
| **Bi-polar evaluation details** |  |
| **Bi-polar evaluation (volts)**  3+2-  2+1-  3+1-  2+0-  3+0-  1+0- | N=25  1.0 (0.6)  0.8 (0.6, 1.3)  N=24  0.9 (0.5)  0.9 (0.6, 1.0)  N=24  0.9 (0.4)  0.9 (0.6, 1.1)  N=24  0.9 (0.4)  0.9 (0.6, 1.2)  N=24  1.0 (0.5)  0.9 (0.7, 1.3)  N=25  1.0 (0.4)  1.0 (0.7, 1.4) |
| **Medtronic evaluation (volts)**  0-3+  1-3+  2-0+  3-0+  0-, 1-3+  1-, 2-3+  2-, 3-0+ | N=12  1.0 (0.6)  0.9 (0.6, 1.5)  N=11  0.8 (0.4)  0.9 (0.5, 1.2)  N=11  0.8 (0.5)  0.9 (0.4, 1.0)  N=11  1.2 (0.4)  1.3 (0.8, 1.4)  N=10  0.9 (0.4)  0.9 (0.7, 1.2)  N=10  0.9 (0.5)  0.9 (0.6, 1.1)  N=10  0.9 (0.5)  0.9 (0.5, 1.1) |

*Footnote: * Denominators may vary for individual measures due to missing data.*

***Supplementary Table 7.*** *Specific reasons for withdrawal from the crossover and cohort follow-up phase.*

| Crossover phase | - no reason given (N =4) - did not want risk of device being switched off (N =3) - SNS removed (N = 2) - loss to follow-up due to COVID-19 (N = 2) - non-compliant with study diaries/visits (N = 1) |
| --- | --- |
| Cohort follow-up phase | - waited too long for appointments due to COVID-19 (N = 1) - loss to follow-up due to COVID-19 (N = 1) - non-compliant with study diaries/visits (N = 1) |

***Supplementary Table 8.*** *Paper bowel diary data completeness for primary outcome (total number of FI episodes).*

| **Time period** | **Number of days paper diary completed for primary outcome (FI events)** | **Randomised allocation** | | **Total**  **n (%)**  **N=39** |
| --- | --- | --- | --- | --- |
|  |  | **SNM/SHAM**  **n (%)**  **N=17** | **SHAM/SNM**  **n (%)**  **N=22** |  |
| **Baseline  (T-18 to T-14)** | **0** | 4 (24) | 2 (9) | 6 (15) |
|  | **1** | 0 (0) | 1 (5) | 1 (3) |
|  | **23** | 1 (6) | 0 (0) | 1 (3) |
|  | **25** | 1 (6) | 1 (5) | 2 (5) |
|  | **26** | 0 (0) | 2 (9) | 2 (5) |
|  | **27** | 1 (6) | 0 (0) | 1 (3) |
|  | **28** | 10 (59) | 16 (73) | 26 (67) |
|  | | | | |
| **Before crossover (T+12 to T+16)** | **0** | 5 (31) | 9 (41) | 14 (37) |
|  | **2** | 1 (6) | 0 (0) | 1 (3) |
|  | **27** | 1 (7) | 0 (0) | 1 (3) |
|  | **28** | 9 (56) | 13 (59) | 22 (58) |
|  | | | | |
| **After crossover (T+28 to T+32)** | **0** | 6 (38) | 15 (68) | 21 (55) |
|  | **19** | 0 (0) | 1 (5) | 1 (3) |
|  | **21** | 1 (6) | 0 (0) | 1 (3) |
|  | **26** | 0 (0) | 1 (5) | 1 (3) |
|  | **28** | 9 (56) | 5 (23) | 14 (37) |
|  | | | | |
| **End of study (T+54 to T+58)** | **0** | 5 (31) | 12 (55) | 17 (45) |
|  | **25** | 1 (6) | 1 (5) | 2 (5) |
|  | **28** | 10 (63) | 9 (41) | 19 (50) |

***Supplementary Table 9.*** *Sensitivity analysis (based on imputation of zero for missing counts when at least one item had been completed per day) on paired t-tests for paper bowel diary outcomes for crossover cohort.*

| **Outcome** | **N=16** | | | |
| --- | --- | --- | --- | --- |
|  | **SNM**  **Mean (SD)**  **Median (IQR)** | **SHAM**  **Mean (SD)**  **Median (IQR)** | **Mean difference* (95% CI)** | ***p^a^*** |
| Number of FI episodes per week (urge + passive) | 2.1 (2.4)  1.4 (0.5, 2.8) | 3.0 (3.7)  1.4 (0.8, 4.3) | -0.9 (-1.8, 0.0) | 0.04 |
| Number of Urgency episodes per week | 3.0 (2.3)  3.1 (1.1, 4.5) | 2.7 (2.6)  1.6 (0.8, 3.8) | 0.3 (-0.6, 1.3) | 0.45 |
| Number of Urge episodes per week | 0.6 (0.8)  0.3 (0.0, 0.9) | 0.9 (1.4)  0.5 (0.1, 1.0) | -0.3 (-0.8, 0.2) | 0.27 |
| Number of Passive faecal incontinence episodes per week | 1.5 (2.3)  0.8 (0.0, 1.4) | 2.1 (3.8)  0.4 (0.0, 1.6) | -0.7 (-1.7, 0.3) | 0.18 |
| Number of wind without control episodes per week | 13.0 (17.3)  4.8 (1.6, 19.1) | 24.7 (38.2)  5.9 (1.4, 31.9) | -11.7 (-25.4, 2.0) | 0.09 |

*Footnote: a: 2-sided p-value from paired t-test. *: direction of difference: SNM-SHAM; hence negative differences indicate fewer episodes with SNM vs SHAM*

***Supplementary Figure 3.*** *Bland-Altman plot of total recorded FI events over the 28-day baseline recording period based on paper diaries vs. e-event recording.*

*Footnote: the wide 95% limits of agreement (-19.7 to + 34 events) exclude only one observation. On average, paper bowel diaries recorded 7.2 more FI events over the 28-day period than the e-event recordings (concordance correlation coefficient = 0.632 [95% CI: 0.37-0.89]).*

***Supplementary Table 10.*** *Perception of group allocation.*

|  | **Randomised allocation** | | | |
| --- | --- | --- | --- | --- |
|  | **SNM/SHAM**  **N*=17** | | **SHAM/SNM**  **N*=22** | |
|  | **Period 1** | **Period 2** | **Period 1** | **Period 2** |
| **Intervention patient believes they have been having since last visit (%)** | N=9 | N=9 | N=5 | N=5 |
| SNM | 6 (67) | 5 (56) | 3 (60) | 3 (60) |
| SHAM | 3 (33) | 4 (44) | 2 (40) | 2 (40) |

*Footnote: * Denominators may vary for individual measures due to missing data.*

***Supplementary Table 11****. Results for symptom questionnaires at baseline and end of study (T58) for those with data available at both timepoints^1^.*

| **Outcomes** | **Baseline** | **End of study (T58)** |
| --- | --- | --- |
| **Summative questionnaire assessments** | | |
| **St Mark’s Incontinence Score^2^** |  |  |
| Original St Mark’s Score  Mean (SD)  Median (IQR) | N=21  19.0 (2.3)  19.0 (18.0, 20.0) | N=21  13.5 (5.7)  16.0 (10.0, 17.0) |
| Modified St Mark’s Score  Mean (SD)  Median (IQR) | N=20  18.4 (2.9)  19.0 (16.5, 20.0) | N=20  12.2 (6.6)  13.0 (7.0, 17.0) |
| **OverActive Bladder symptoms (OAB-Q) short form^3^** |  |  |
| OAB-Q SF score  Mean (SD)  Median (IQR) | N=23  31.7 (23.2)  26.7 (13.3, 53.3) | N=23  30.4 (20.3)  30.0 (13.3, 46.7) |
| **Fecal Incontinence Quality of Life (FI QOL)^4^** |  |  |
| Lifestyle mean score  Mean (SD)  Median (IQR) | N=22  2.0 (0.6)  1.8 (1.6, 2.5) | N=22  2.8 (0.9)  3.0 (2.4, 3.6) |
| Coping behaviour mean score  Mean (SD)  Median (IQR) | N=17  1.3 (0.4)  1.2 (1.0, 1.4) | N=17  2.4 (0.7)  2.3 (2.1, 2.9) |
| Depression/self-perception mean score  Mean (SD)  Median (IQR) | N=16  2.0 (0.4)  2.0 (1.8, 2.3) | N=16  2.7 (0.7)  2.5 (2.3, 3.4) |
| Embarrassment mean score  Mean (SD)  Median (IQR) | N=23  1.7 (0.8)  1.3 (1.0, 1.7) | N=23  2.2 (1.0)  2.0 (1.3, 2.7) |
| **International Consultation on Incontinence Bowel (SF-ICIQ-B) questionnaire^5^** |  |  |
| SF-ICIQ-B mean score  Mean (SD)  Median (IQR) | N=24  9.0 (1.1)  9.3 (8.5, 9.9) | N=24  7.1 (2.4)  7.8 (5.4, 8.8) |
| **EuroQol Health Outcome Measure (EQ-5D-5L)** |  |  |
| Summary index value^6^  Mean (SD)  Median (IQR) | N=22  0.7 (0.2)  0.8 (0.7, 0.9) | N=22  0.8 (0.2)  0.8 (0.7, 0.9) |
| EQ-VAS score^7^  Mean (SD)  Median (IQR) | N=23  66.1 (18.2)  65.0 (50.0, 80.0) | N=23  72.9 (19.9)  80.0 (50.0, 90.0) |
| **Likert scale of patient’s global impression of treatment success** | - |  |
| Satisfaction score^8^  Mean (SD)  Median (IQR) | - | N=24  73.8 (20.8)  80.0 (65.0, 87.5) |

*Footnote: ^1^ Denominators may vary for individual questionnaire measures due to missing data at one or both of the timepoints. ^2^ For both the modified and original St Mark’s scores, the minimum score is 0 and the maximum total score is 24; high scores indicate more incontinence. ^3^ The OAB-Q short form score has a range of 0-100; higher scores indicate greater symptom bother. ^4^ Each FI QOL subscale score ranges from 1 to 4; higher scores indicate greater quality of life. ^5^ SF-ICIQ-B mean score has a range of 0-10; higher scores indicate greater importance of bowel incontinence on life issues. ^6^ Summary index values will be presented against country specific value sets. ^7^ VAS records the respond’s self-rated health on a scale from 0 to 100; higher scores indicate impressions of better health. ^8^ Satisfaction score indicates the extent to which the patient feels that their symptoms have improved compared with before the study. The score ranges from 0%=not at all to 100%=complete cure.*

***Supplementary Table 12.*** *Adverse events.*

|  | **N=10** | **Allocation at time of AE/SADE** | **Severity** | **Causality** | **Outcome** | **Expectedness** |
| --- | --- | --- | --- | --- | --- | --- |
| **SADE** |  |  |  |  |  |  |
| Fall leading to lead migration and device deficiency requiring revision | 1 | SHAM | severe | definite | resolved | unexpected |
| **AE** |  |  |  |  |  |  |
| Ache at IPG site* | 1 | SHAM | mild | probable | unresolved | expected |
| Cramp in leg | 1 | SNM | mild | unlikely | resolved | unexpected |
| Pain in right abdominal area | 1 | SNM | mild | unlikely | resolved | unexpected |
| Wound infection | 1 | SHAM | mild | definite | resolved | expected |
| Diarrhoea and abdominal pain | 1 | SNM | moderate | possible | resolved | unexpected |
| Urinary tract infection | 1 | SNM | mild | possible | resolved | unexpected |
| Stress urinary incontinence | 1 | SNM | moderate | possible | resolved | unexpected |
| Shooting pains in leg | 1 | SHAM | mild | unlikely | resolved | expected |
| Numbness since insertion | 1 | SHAM | moderate | possible | resolved | unexpected |

*Footnote: SADE: serious adverse device-related event; AE: adverse event; IPG: implanted pulse generator; *patient complained of mild ache towards end study participation. The device was eventually removed after study completion.*
